# Long-term obesogenic diet leads to metabolic phenotypes which are not exacerbated by catch-up growth in zebrafish

**DOI:** 10.1371/journal.pone.0267933

**Published:** 2022-05-11

**Authors:** Sandra Leibold, Amrutha Bagivalu Lakshminarasimha, Felix Gremse, Matthias Hammerschmidt, Maximilian Michel

**Affiliations:** 1 Institute of Zoology, University of Cologne, Cologne, Germany; 2 Cologne Excellence Cluster on Cellular Stress Responses in Aging-Associated Diseases, University of Cologne, Cologne, Germany; 3 Department of Nanomedicine and Theranostics, Institute for Experimental Molecular Imaging, Faculty of Medicine, RWTH Aachen University, Aachen, Germany; 4 Gremse-IT GmbH, Aachen, Germany; 5 Center for Molecular Medicine Cologne, University of Cologne, Cologne, Germany; Medical University of Vienna, AUSTRIA

## Abstract

Obesity and metabolic syndrome are of increasing global concern. In order to understand the basic biology and etiology of obesity, research has turned to animals across the vertebrate spectrum including zebrafish. Here, we carefully characterize zebrafish in a long-term obesogenic environment as well as zebrafish that went through early lifetime caloric restriction. We found that long-term obesity in zebrafish leads to metabolic endpoints comparable to mammals including increased adiposity, weight, hepatic steatosis and hepatic lesions but not signs of glucose dysregulation or differences in metabolic rate or mitochondrial function. Malnutrition in early life has been linked to an increased likelihood to develop and an exacerbation of metabolic syndrome, however fish that were calorically restricted from five days after fertilization until three to nine months of age did not show signs of an exacerbated phenotype. In contrast, the groups that were shifted later in life from caloric restriction to the obesogenic environment did not completely catch up to the long-term obesity group by the end of our experiment. This dataset provides insight into a slowly exacerbating time-course of obesity phenotypes.

## Introduction

Metabolic syndrome is becoming an increasingly prevalent global health concern. Metabolic syndrome is an umbrella term used for various pathologies surrounding long-term obesity such as hypertension or type 2 diabetes [1] and whether an individual develops obesity and metabolic syndrome is a complex interplay of genetic and environmental contributions. While studies suggest that heritable factors account for 40% to 85% of the variation in adiposity [2] the environmental contribution is still immense, even at birth [3–5]. Maternal body weight and overnutrition during the gestational period have been implicated in increasing the risk of metabolic syndrome for offspring [5, 6]. Further, high birth weight and/or rapid infant growth [7, 8] as well as low birth weight / pre-term birth and a period of subsequent catch-up growth [9–11] are associated with an increased risk of metabolic syndrome later in life. Evidence in rodents shows that a period of early food restriction or malnutrition—especially in the peri-natal period–followed by compensatory growth is correlated with metabolic risk in later life [9, 12]. Caloric restriction leads to growth stagnation or reduced growth and when conditions are favourable of growth again, animal growth increases again. This phenomenon has been most frequently referred to as “compensatory growth” [13] and can be observed across vertebrates including people [1, 14], livestock [15, 16], amphibians [17], birds [18], reptiles [19, 20] or fish [21, 22]. However, an important distinction is that differences can be seen in the growth rate of the organism after the growth restricting conditions are alleviated. The increased growth is called catch-up growth if it occurs at the same growth rate as matched (size or age) animals that were never restricted; or compensatory growth if growth rates are higher than in matched animals [13]. Importantly, in mammals, compensatory responses with above normal growth rates are associated with altered body composition later in life [23], particularly after prenatal [24, 25] or early postnatal food restriction [26, 27], while catch-up growth has not been associated with long-term detrimental outcomes. However, the effect of compensatory of catch-up growth (CG) on long term metabolic outcomes is controversial in children, and it has been argued that when measured as a matter of gestational age, the effect of birthweight on long-term metabolic outcomes disappears and therefore the long-term detrimental outcomes have potentially more to do with premature birth (and therefore low birthweight) than with low birthweight in babies carried to term [1].

Zebrafish have emerged as an important vertebrate model system for several reasons. Not only are genes largely conserved but there is also a high degree of physiological and anatomical similarity. Genetic models of obesity have been established in zebrafish such as overexpression of agouti related peptide [28] or semaphorin 3 [29, 30]. Metabolically, zebrafish show evidence for metabolic syndrome endophenotypes such as diet induced obesity, diabetes (reviewed in [31], dyslipidemia (reviewed in [32], non-alcoholic or alcoholic fatty liver disease (reviewed in [33, 34]. Further, key obesity hallmarks have been identified in zebrafish downstream of overfeeding. Obesity is associated with increased body growth due to overfeeding [35–38] as well as increased lipid deposition in subcutaneous and visceral adipose depots [37, 38]. However, to long-term detrimental effects of long-term obesity are not entirely clear in people [39, 40] let alone in fish. However, recent studies in fish support that obesity also leads to long-term effect in fish such as cognitive decline and inflammation of the central nervous system [41, 42]. Peripherally, it has been shown histologically that adipocytes not only undergo hypertrophy but also hyperplasia in zebrafish exposed to an obesogenic diet [36]. Hyperplasia in mammals is considered to be an adaptive response allowing the safe storage of fat while subcutaneous adipocyte hypertrophy is correlated with metabolic complications [43]. Additionally, fish store triglycerides not only in fat but also in muscle and liver under normal conditions [44], so increased hepatic lipid metabolism [37] or deposition [38] could be normal. Abnormalities consistent with hepatic steatosis have also been seen in DIO zebrafish [35, 45, 46]. A reduced metabolic rate has been linked to metabolic syndrome in people [47, 48], however this association has been questioned in mouse model studies [49–51]. In zebrafish, a recent study showed an increased metabolic rate as well as mass for overfed fish [52], suggesting that the effect may be driven by mass rather than a decreased energy expenditure in the obese animals. Similarly, obesity and in particular insulin resistance have been linked to functional changes in mitochondria in people [53] as well as mice [54, 55]. In zebrafish, mitochondrial function has to our knowledge only been looked at in the context of a CG response, where hepatic oxidative phosphorylation was shown to be enhanced in animals undergoing CG [56] but not in the context of obesity. Lastly abnormalities in glucose metabolism particularly hyperglycemia, impaired glucose tolerance and insulin resistance are hallmarks of type 2 diabetes, a condition intricately linked to metabolic syndrome and have been described in mammals [1], as well as fish [38, 57, 58].

Consequently, a variety of metabolic complications due to an obesogenic environment have been described in zebrafish. Further, a recent study in zebrafish has found that maternal overfeeding has a negative effect on early obesity markers in larvae of the next generation, comparable to evidence in mammals [42]. Therefore, zebrafish provide an elegant system to study the basic biology of energy homeostasis. However, it is unclear whether early undernutrition followed by CG could exacerbate markers of obesity—even though CG is a well-studied phenomenon in fish (reviewed in [22].

Consequently, we carried out a systematic characterization of DIO as well as CG. We compared CR with DIO and tested whether CG of fish which were shifted from CR to DIO at different timepoints showed an exacerbated long-term DIO phenotype. Zebrafish are externally fertilized and feed for the first 5 days-post fertilization (dpf) on maternally provided egg yolk. We therefore restricted food from 5dpf onwards and induced CG by overfeeding comparable to the DIO group. We induced CG at three timepoints associated with different growth rates in wildtype animals: 1) a juvenile phase (one month) which exhibits naturally high growth rates (CG1), 2) three months old fish, a stage when growth begins to slow (CG3) and 3) nine months of age when growth mostly stagnates in wildtype fish (CG9). We investigate growth parameters, fat distribution and metabolic genes in order to establish whether zebrafish a) show signs of compensatory or catch-up growth, b) show signs of metabolic syndrome after a long-term obesogenic diet in support of and beyond previous studies and c) whether early food restriction exacerbates the effects of long term DIO and pre-disposes fish for or exacerbates metabolic syndrome.

## Materials and methods

### Zebrafish maintenance

Zebrafish of the Ekkwill strain were raised and maintained at 28°C under a 14 h light / 10 h dark cycle and in tanks with continuous water exchange and recycling (AquaSchwarz, Göttingen, Germany) [59]. Embryos were obtained by natural mating while siblings derived from the same mating of a single parental pair were used for comparative analyses of aged-matched fish.

Fish were raised as previously reported in groups of 5 (DIO, CG after induction) or 50 fishes (CR, CG before induction) in 3 L tanks [36]. In our previous publication, the DIO condition was labelled as “high amounts of food, low density” (HF-LD) and the CR condition was called “low amounts of food, high density” (LF-HD). Compensatory growth was induced by splitting one tank with 50 fish in 10 tanks containing 5 fish each. Fish were sorted for sex as soon as visible and maintained at a female:male ratio of 3:2. Body length of 10 fish of each group was measured at least every other week and density was checked weekly. Number and sex ratio of each tank were checked before each measurement but at least once per month.

We carried out a control experiment for CG1 where we compared changing the amount of food at a constant density versus changing density at constant food levels (S2 Fig). For this, we fed as follows.

Changing food levels, constant density: 5 fish per 2.5 liter (L) fed either

1. DIO: 100 ml paramecia daily (5–13 days post fertilization (dpf)) and 2x 10 drops of artemia (14 dpf onwards).
2. CR: daily feeding of 10 ml paramecia (5–13 dpf) and 2x 1 drop of artemia (14 dpf onwards).
3. CG1: CR conditions from 5-30dpf, DIO conditions 30dpf and older

Constant food levels, changing density: feeding under DIO conditions as above, density either

1. DIO: 5 fish per 2.5L tank for the extent of the experiment
2. CR: 50 fish per 2.5L tank for the extent of the experiment
3. CG1: 50 fish per 2.5L tank from 5dpf until 30dpf which were split into 10 times 5 fish per 2.5L tank at 30dpf onwards

All zebrafish experiments were approved by the national animal care committee (LANUV Nordrhein-Westfalen; 8.87–50.10.31.08.130; 84–02.04.2012.A253) and the University of Cologne. Animals were sacrifices with an overdose of Tricaine MS-222 according to [60].

### Determination of body length and body weight

For determination of body length and body weight, fish were anesthetized in 0.13% Tricaine (w/v). Body length was measured from the anterior tip of the mouth to the base of the caudal fin (standard length, SL) using millimeter paper (to 0.5 mm). For determination of body weight, anesthetized fish were dried on Whatman paper and measured to one decimal point in milligrams. Per measurement 10 fish (as soon as visible 5 males and 5 females) were measured individually per condition.

### Calculation of specific growth rate

We calculated specific growth rate (SGR) in %weight per day according to [61] calculation 9 which states SGR = (Ln(weight 2)-Ln(weight 1))/(Day 2—Day 1) x 100 where day 1 and weight 1 is the first measurement and day 2 and weight 2 the subsequent measurement.

### RNA extraction and quantitative real-time PCR

Total RNA was purified from pool of 3 brains or individual bodies with the PureLink RNA Mini Kit (life technologies, Carlsbad, USA) after homogenization of the frozen material with mortar and pestle on liquid nitrogen and RNA extraction with Trizol (Invitrogen, CA, USA). cDNA was synthesized with Superscript II Reverse Transcriptase (Invitrogen, CA, USA) according to the manufactor`s instructions.

Quantitative real-time PCR (qRT-PCR) was performed in quadruplicates with an Applied Biosystems 7500 Fast Real-Time PCR system (Applied Biosystems, Foster City, USA), SYBR Select Master Mix (Applied Biosystems, Foster City, USA) and primers for *agrp* [62] and *insa* [63]. Relative mRNA expression levels were determined with Biosystems Prism SDS and Excel software, using the expression level of *rps23* [64] as an internal standard.

### Quantification of food intake

After determination of body weight, fish were put in single tanks (1 L) to acclimate and were starved for at least 24 hours. Feeding experiments took place at room temperature in a separated room without any disturbance to avoid stressing the fish. At least 5 fish were tested per condition.

Because there is no standard method available for quantification of food intake in adult zebrafish, a new method for quantification of food intake in adult zebrafish was established. Instead of commercial fish food, living third instar larvae of wild-type *Drosophila melanogaster* (*D*. *melanogaster*) were used for quantification of food intake because they can survive several hours in water without changing weight and keep moving making them an attractive prey for zebrafish. *D*. *melanogaster* provided by the Roth laboratory at the University of Cologne were used to raise larvae for the feeding experiment. Third instar larvae of *D*. *melanogaster* were washed out of the feeding medium, counted, dried using Whatman paper and weighted. Larvae with a weight of at least 10% of the fish`s body weight were given to the fish. During the following 8 hours, every 1–2 hours a counted number of fresh larvae were given to the fish. After one and after eight hours, fish were put in single tanks with fresh water and remaining larvae were counted and weighted. Weight was measured to one decimal points in mg using a precision scale. Food intake during the first hour was calculated in fed larvae in % bodyweight, while food intake during eight hours (whole day) was calculated in fed larvae/100 mg body weight.

### μCT analysis

For μCT imaging, adult zebrafish were fixed and decalcified in Bouin`s solution at room temperature for 7 days, stored in PBS and imaged using a micro-computed tomography (μCT) device (SkyScan1272, Bruker BioSpin GmbH, Ettlingen, Germany). Zebrafish were placed individually in 1.5ml Eppendorf tubes using and an ultra-focus scan over the whole body was performed in a full-rotation in step-and-shoot mode. 322 projections (1008x672 pixels, 4x4 binning) were acquired per subscan with an x-ray tube voltage of 60 kV, power 0.166 mA, aluminum filter 0.25 mm,exposure time of 363 ms, 6 averages and a object-source distance of 86 mm. All CT images were reconstructed at an isotropic voxel size of 18 μm using a Feldkamp type algorithm (filtered back-projection).

Fat-containing regions were appear hypo intense in μCT data and were segmented using Imalytics Preclinical (Gremse-IT GmbH, Aachen, Germany [65]. The volumetric fat percentage was calculated as the ratio of subcutaneous adipose tissue (SAT) or visceral adipose tissue (VAT) fat volume compared to the entire volume of the body cavity anterior of the anal fin and expressed per skeletal segment.

### Histology

For histological analyses, adult zebrafish were fixed and decalcified in Bouin`s solution at room temperature for 7 days and stored. After μCT imaging, male fish were fixed in 4% PFA overnight at 4°C followed by PBS washes the next day. Viscera were removed, livers dissected from the fixed samples and oriented in 3% agarose in 30% sucrose/PBS and mounted in tissue freezing medium (Leica). The head and tail regions were removed by cutting out the tissue posterior to the operculum and anterior to the dorsal fin. The rest of the trunk (without the viscera) was subsequently bisected, decalcified in 0.5 M EDTA, embedded in the same way as mentioned for the livers. 8–10 μm transverse sections of the trunk and livers were obtained using a Leica CM1850 cryostat. The slides containing sections were air dried for 60 minutes at room temperature and fixed in ice cold 4% PFA for 10 minutes. After fixation, the samples were washed thrice in PBS for 5 minutes each and treated with 100% propylene glycol for 5 minutes. Meanwhile, the Oil Red O (ORO) (Sigma-Aldrich) stock solution (0.5g Oil Red O in 100 ml propylene glycol) was filtered and prewarmed at 60°C. The samples were stained with ORO for 8–10 minutes at 60°C. The slides were differentiated in 85% propylene glycol for 5 minutes, rinsed twice in distilled water and counterstained with Gill’s haematoxylin for 30 seconds. Excess stain was removed by washing with running tap water for a minute, rinsed in distilled water and mounted with Aqua-Mount® (Avantor).

For the quantification of subcutaneous adipocytes, ORO and haematoxylin-stained transverse sections of three levels were selected, total numbers and areas per side around the pleural ribs (PR) region was measured using ImageJ. Average adipocyte cell size along both sides of the pleural rib in each section was calculated using area divided by cell number. The single adipocyte cell sizes were further validated using ImageJ. For each condition, two fish and three consecutive sections were analysed, and the mean was recorded.

#### Analysis of oxygen consumption

After determination of body weight, fish were put in single tanks (1 L) to acclimate and were starved for at least 24 hours. Determination of oxygen consumption was performed at room temperature (19–20°C) in a separated dark room without any disturbance to avoid stressing the fish. Individual fish were placed in respiratory chambers and allowed to acclimate for at least 30 min with continuous water flow before measurements started.

The experimental set-up consisted of 4 respiratory chambers (Loligo Systems, Tjele, Denmark; for adult fish: diameter = 40 mm, volume = 38 ml) with ministirrers inside which are placed in a water bath. Fresh air-bubbled water is provided using a Minipuls 3 flushing pump (Gilson, Limburg-Offheim, Germany) which is controlled by a computer. Dissolved oxygen levels were measured using Oxy 4 mini (Presens GmbH, Regensburg, Germany), a multichannel oxygen measuring system which connected via optical cables with oxygen sensors (Presens GmbH, Regensburg, Germany) which are glued inside the chambers. Oxygen levels were recorded every second during a 5 min phase in each interval (20 min flushing, 15 min waiting, 5 min measuring). Flushing is sufficient to exchange more than 99% of the water [66]. Oxygen sensors and optic fibres were calibrated using air (100% air saturation) and nitrogen bubbled water (0% air saturation) while a temperature probe was calibrated using ice (0°C) and boiling water (100°C) as described in the manual. Calibrations were done prior to each experiment. Oxygen consumption was calculated using the programme AutoResp2 (Loligo Systems, Tjele, Denmark) and determined during at least three days (72 h). Measured values with the coefficient of determination (R^2^) > 0.9 were used to calculate routine and standard metabolism. Mean of oxygen consumption rate during 24 hours was defined as routine metabolism [67], while mean of the 5 lowest oxygen consumption rates were assumed to reflect SMR [68, 69]. Both rates were expressed as mg O_2_ / kg/ h.

### Analysis of mitochondrial function using respirometry

After determination of body length and body weight, fish were sacrificed, cut in small pieces and digested with trypsine on ice for 40 min. Intact mitochondria were isolated according to [70]. The mitochondrial protein concentration was determined using a Bradford assay.

Oxygen consumption rates were measured with Oxygraph-2k (Oroboros Instruments, Innsbruck, Austria) at 28°C using 150–200 μg crude mitochondria as described before [71]. In short, mitochondrial complex I activity was measured with the addition of 1 mM ADP, 5 mM pyruvate, 2 mM malate, and 10 mM glutamate (maximal rate of oxidative phosphorylation of complex I, OXPHOS CoI). Then, mitochondrial coupling was evaluated by the inhibition of ATP synthase by adding 1.5 mg/ml oligomycin and uncoupling by a multiple-step carbonylcyanide p-trifluoromethoxyphenylhydrazone titration (membrane leakage, LEAK). Afterwards the uncoupler FCCP is added till a maximal plateau is reached to determinate maximal oxygen consumption flux due to electron transfer system (ETS). By adding 0.5 mM rotenone, complex I is inhibited to assess oxygen consumption flux due to reactive oxygen species (ROX). Of note, oxygen concentration below 100 nmol/ml were avoided.

### Analysis of blood glucose levels in adult zebrafish

Blood sugar levels were determined using the glucose meter Accu-Chek Aviva (Roche Diagnostics, Risch, Switzerland) as described [72]. In short, fish were anesthetized in ice for 60 seconds and sacrificed afterwards by decapitation. The cut was immediately anterior to the articulation of the pectoral fin with the girdle, and severed the heart. Whole blood was analysed immediately by applying a test strip directly to the cardiac blood.

### Statistical analysis

Statistics were carried out in GraphPad Prism version 8.4.3 for Windows, GraphPad Software, San Diego, California USA. The particular test used is indicated in each section. P-values less than 0.05 were considered significant.

### Primary data

We have made the primary data available through GFBio [73] and the primary data is available under https://doi.org/10.1594/PANGAEA.941313 while the CT scans are available under https://doi.org/10.1594/PANGAEA.940201.

## Results

### CG in zebrafish—characterization

**Fig 1:**
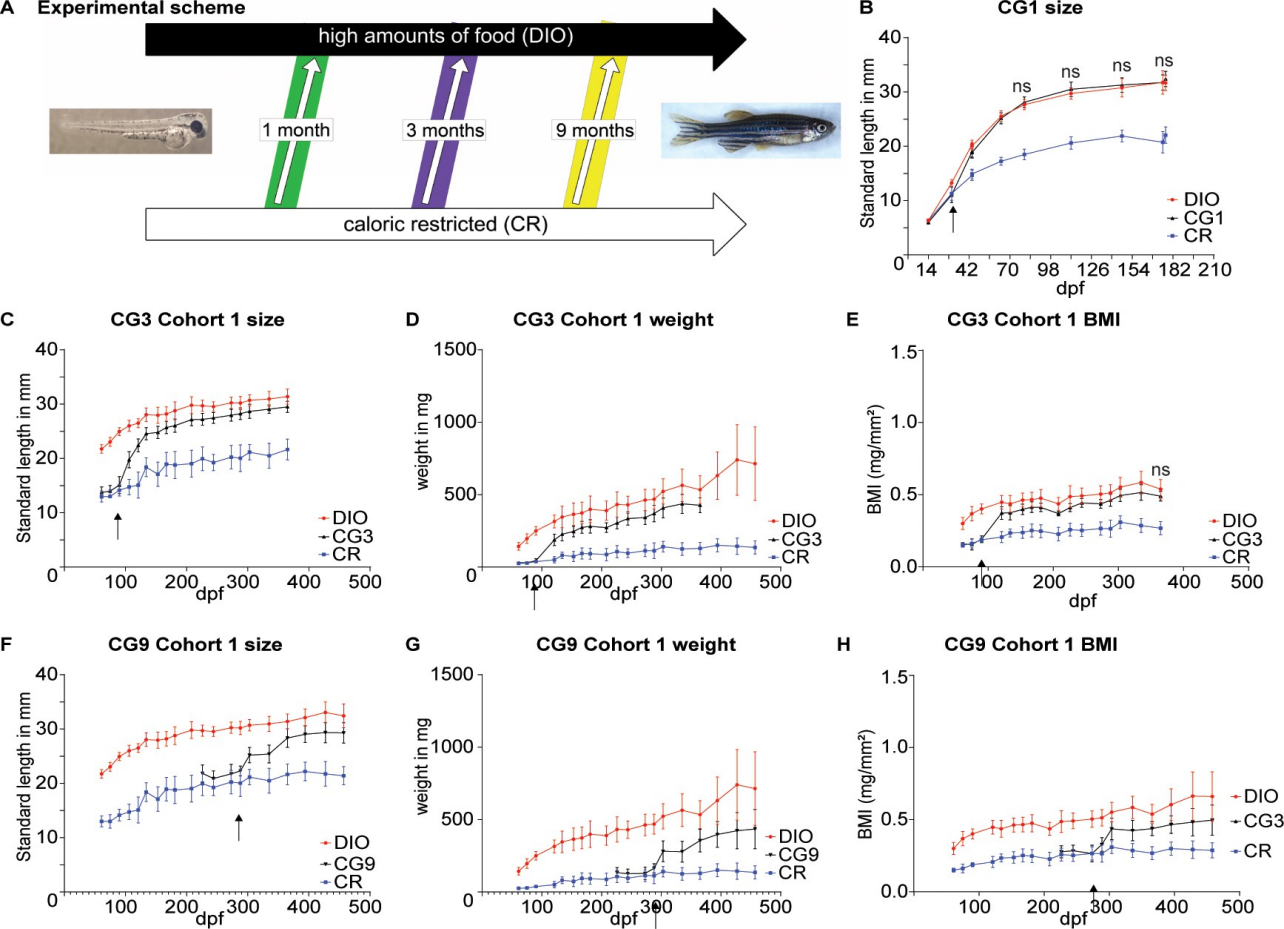
Compensatory growth can be induced in juvenile and adult zebrafish by excessive feeding following caloric restriction and results in compensation for differences in body length and BMI. (A) Scheme showing changes in feeding regime to induce compensatory growth (CG) in zebrafish with an age of one, three or nine months; (B) Standard length of fish undergoing CG at 1 month of age (arrow); (C, F): Standard Length; (D, G) body weight; (E, H) and body mass index of a cohort of fish (n = 10) undergoing CG at 3 months of age (C-E) and at 9 months of age (F, H); error bars indicate STDEV, ns indicates a lack of significant difference between the CG and the DIO group at that timepoint as indicated by a 2-Way ANOVA followed by Tukey’s multiple comparison test.

We compare calorically restricted (CR) fish with overfed / diet induced obese (DIO) fish and fish that were shifted from CR to an obesogenic environment after 1, 6 or 9 months of CR (compensatory or catch-up growth; CG1, CG3 and CG9 respectively, see also Fig 1A and 1B).

Consistent with previous work, overfeeding leads to increased somatic growth [35, 36]. We carried out multiple 2-Way ANOVA which in all cases showed a significant interaction of time (fish grow longer and heavier over time) and food (DIO fish grow faster than CR fish). For all datasets, CG fish started growing significantly when transferred to the obesogenic environment. We were interested in whether growth approaches the DIO curve and therefore only indicate on the graphs where the difference between the DIO and the CG group is not significantly different anymore according to Tukey’s multiple comparison test.

For the early CG1 group we measured only the standard length. DIO fish grow significantly faster than CR fish. After transfer of the CG1 fish from CR to DIO conditions, fish compensate quickly. While we still saw a significant difference between DIO and CG1 at 44 and 64dpf, the two groups are not significantly different from 80 days onwards (Fig 1B; indicated as not significantly different between CG1 and DIO as indicated by a 2 Way ANOVA MCT; effect of time F(9, 252) = 1301, p<0.0001; effect of food F(2, 252) = 1095, p<0.0001; interaction F(18, 252) = 40.18, p<0.0001). When we induced CG at 3 months of age (CG3), we similarly saw that DIO fish grow faster than CR fish in terms of standard length (Fig 1C; 2 Way ANOVA effect of time F(16, 451) = 175.6, p<0.0001; effect of food F(2, 451) = 2014, p<0.0001; interaction F(32, 451) = 10.96, p<0.0001); body weight (Fig 1D; 2 Way ANOVA effect of time F(15, 424) = 93.75, p<0.0001; effect of food F(2, 424) = 1291, p<0.0001; interaction F(30, 424) = 10.26, p<0.0001) as well as body mass index (BMI, Fig 1E; 2 Way ANOVA effect of time F(15, 424) = 79.29, p<0.0001; effect of food F(2, 424) = 1032, p<0.0001; interaction F(30, 424) = 7.508, p<0.0001). During the compensatory response, CG3 fish showed an initial fast growth followed by a plateau that slowly approached the DIO group but in the time we measured remained significantly different to the DIO group in the two way ANOVA MCT.

When we induced CG at 9 months of age we found a comparable result in that the CG group never quite reached the DIO groups growth, either for standard length (Fig 1F; 2 Way ANOVA effect of time F(9, 265) = 35.09, p<0.0001; effect of food F(2, 265) = 900.4, p<0.0001; interaction F(18, 265) = 7.444, p<0.0001), fish weight (Fig 1G; 2 Way ANOVA effect of time F(9, 265) = 23.10, p<0.0001; effect of food F(2, 265) = 511.2, p<0.0001; interaction F(18, 265) = 4.511, p<0.0001) or BMI (Fig 1H; 2 Way ANOVA effect of time F(9, 265) = 17.26, p<0.0001; effect of food F(2, 265) = 363.1, p<0.0001; interaction F(18, 265) = 3.285, p<0.0001).

We repeated the experiment in a separate cohort with a comparable result–the CG group responded with a period of fast growth and never quite approached growth in the DIO group except in one timepoint (S1 Fig). We again induced CG at 3 months of age and measured standard length (S1A Fig; 2 Way ANOVA effect of time F(11, 324) = 206.9, p<0.0001; effect of food F(2, 324) = 1050, p<0.0001; interaction F(22, 324) = 14.44, p<0.0001), body weight (S1B Fig; 2 Way ANOVA effect of time F(11, 324) = 57.69, p<0.0001; effect of food F(2, 324) = 351.3, p<0.0001; interaction F(22, 324) = 7.463, p<0.0001) and BMI (S1C Fig; 2 Way ANOVA effect of time F(11, 324) = 51.61, p<0.0001; effect of food F(2, 324) = 357.8, p<0.0001; interaction F(22, 324) = 5.066, p<0.0001). For CG9 we also measured standard length (S1D Fig; 2 Way ANOVA effect of time F(10, 297) = 37.99, p<0.0001; effect of food F(2, 297) = 1259, p<0.0001; interaction F(20, 297) = 9.571, p<0.0001), fish weight (S1E Fig; 2 Way ANOVA effect of time F(10, 297) = 15.28, p<0.0001; effect of food F(2, 297) = 464.4, p<0.0001; interaction F(20, 297) = 2.251, p = 0.0019) and BMI (S1F Fig; 2 Way ANOVA effect of time F(10, 297) = 14.61, p<0.0001; effect of food F(2, 297) = 358.1, p<0.0001; interaction F(20, 297) = 1.661, p = 0.0389).

We previously showed that growth at different densities is equivalent to overfeeding at the same density [36]. However, it is conceivable that CG would be impacted in a different way by density changes. Therefore, we also tested the 1month CG condition keeping the number of fish per tank consistent between the CR, CG and DIO groups but varying the food given. We saw no difference in CG between changes in feeding regime with a constant density (S2A Fig; 2 Way ANOVA effect of time F(6, 179) = 198.5, p<0.0001; effect of food F(2, 179) = 339.3, p<0.0001; interaction F(12, 179) = 16.82, p<0.0001) and constant feeding regime with changes in density S2B Fig; 2 Way ANOVA effect of time F(6, 186) = 180.6, p<0.0001; effect of food F(2, 186) = 463.8, p<0.0001; interaction F(12, 186) = 16.13, p<0.0001). If anything, the change in density lead to a faster catch-up period than the change in feeding regime.

### CG in zebrafish–catch up growth in the absence of compensatory growth

In order to establish the mode of growth in zebrafish, we compared growth trajectories. Zebrafish show a typical sigmoidal growth curve. We started measuring too late to see the exponential phase of growth, but in every group, we saw a clear segment of linear growth. Puberty onset occurs at an SL of around 18mm [74] which in the DIO group occurs around 45 dpf and in the CR group occurs around 3 months of age. A decline in growth rate is seen in many fish after sexual maturity [13], and particularly in the DIO curve, fish growth is linear until shortly after maturity and starts becoming asymptotic around 20-25mm SL (Fig 1C, 1D, 1F, 1H and 1J)). Looking at the growth curves, we noticed that CG fish grow in a similar manner to DIO fish of a comparable SL when they are transferred to *ad libitum* conditions, suggesting that fish resume a normal growth trajectory after the period of growth stagnation, which suggests catch-up growth and not compensatory growth.

**Fig 2:**
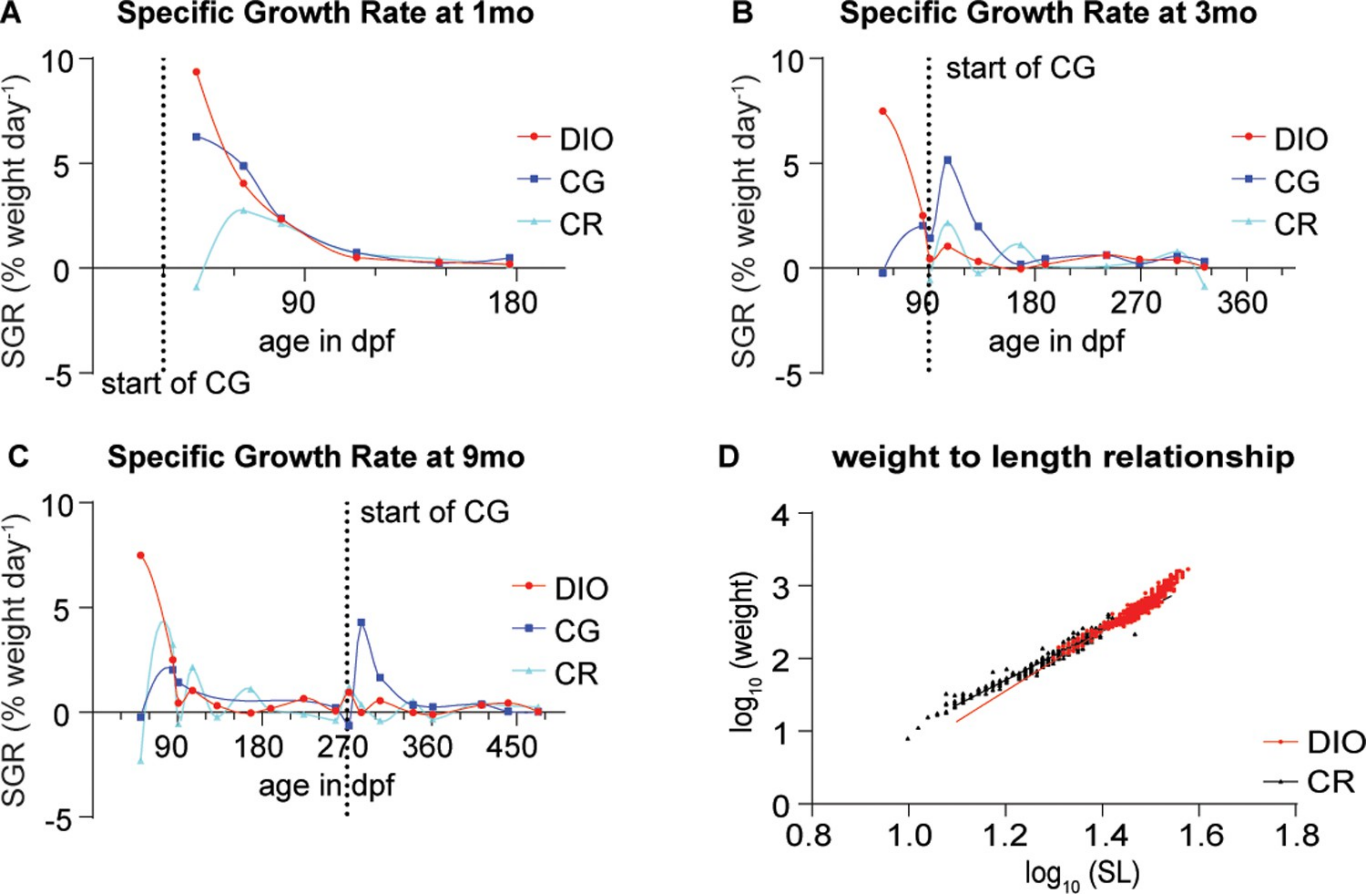
Growth rate and weight-length relationship in fish growing under different conditions. (A-C) Specific Growth rates (SGR) of fish fed under DIO, CG and CR conditions and undergoing CG at (A) one month of age, (B) 3 months of age (C) or 9 months of age. Data fit with an Akima spline curve; (D) The weight-length relationship of CR and DIO fish. The slope is significantly larger in the case of the DIO group compared to the CR group F(1, 970) = 133, p<0.0001.

We calculated growth rates according to [61]. The results did not differ between the cohorts so we only show the results for the second cohort of fish we raised. The growth rate of the DIO fish is the highest during the period of linear growth and rapidly declines during the period of asymptotic growth around 45–90 days followed by a very low residual growth rate (Fig 2A–2C). The growth rate in the CG groups peaks briefly after release from the growth restricting conditions and indeed during this time is significantly higher than the DIO or CR group. Importantly however, the growth rate of the CG group does not rise above the highest growth rate seen in the DIO group at an earlier timepoint, providing evidence that the observed growth is catch-up growth [13].

We also looked at the weight-length relationship for CR and DIO fish with a linear regression analysis of the log weight to log standard length relationship. For DIO fish we found that log(DIO weight) = 4.134*log(SL) -3.404, R^2^ = 0.9037 and for CR fish that log(CR weight) = 3.363*log(SL) - 2.329, R^2^ = 0.9575 (Fig 2D). Therefore, we measure b at 4.134 for DIO fish and 3.363 for CR fish which provides evidence for positive allometric growth in zebrafish (b>3 according to [75]).

### CG in zebrafish—CR and early CG fish are hyperphagic

**Fig 3:**
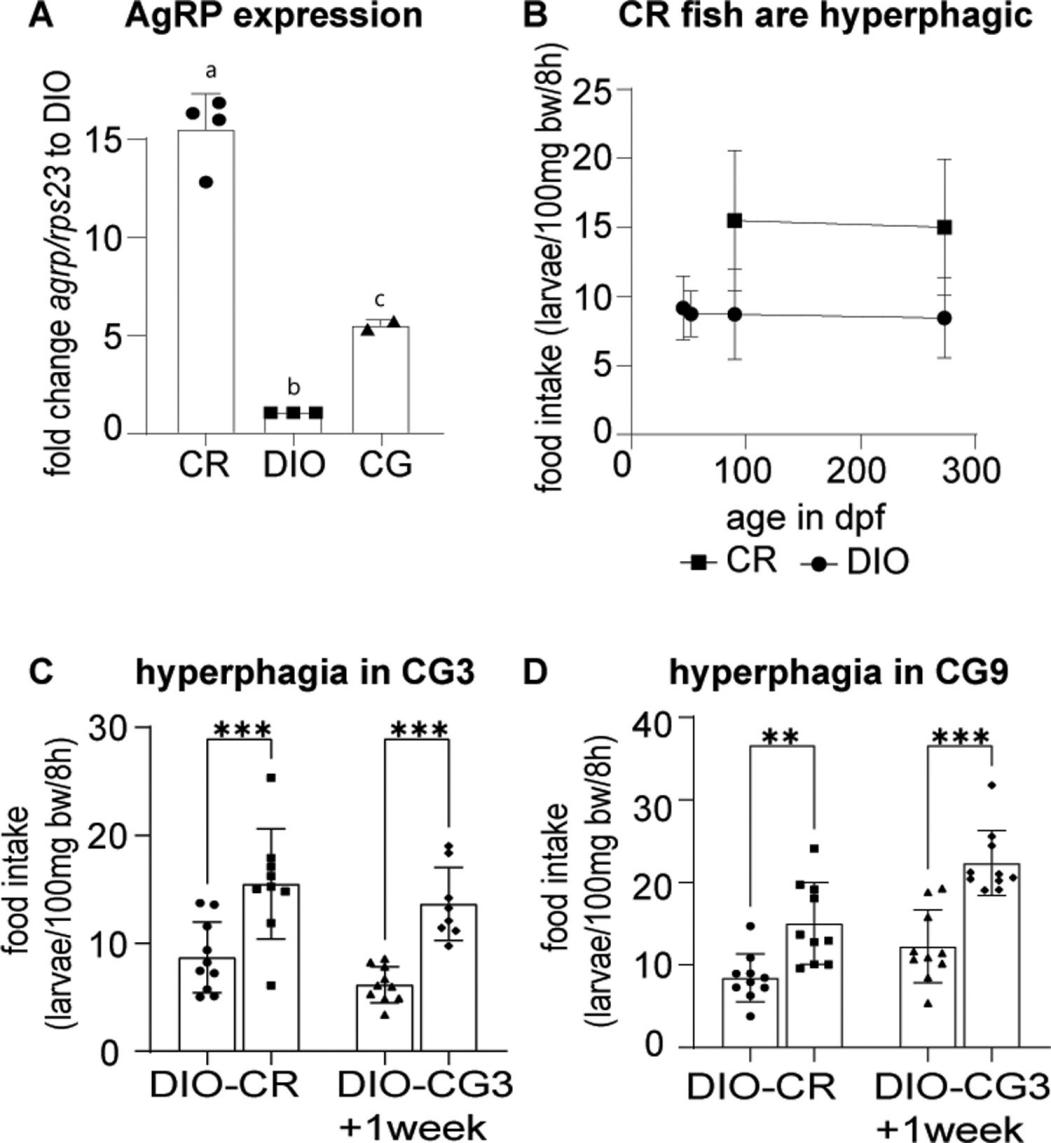
Fish undergoing CR and CG are hyperphagic. (A) *agrp* expression analysis of CR and DIO fish brains as well as CG9 fish brains 1–2 weeks after the shift to the obesogenic environment. Expression is standardized to *rps23*; (B) calorically restricted fish are severely hyperphagic when exposed to *ad libitum* feeding conditions compared to the DIO group that has been raised in an obesogenic environment; (C) CG3 fish are hyperphagic before (CR) transfer to DIO conditions as well as at least one week after transfer (CG3); (D) The CG9 group is similarly hyperphagic before as well as at least one week after the transfer to DIO conditions. Pooled data of male and female fish, n of 4–10; error bars indicate STDEV, groups were compared with ANOVA followed by Tukey’s multiple comparisons test (A) or an unpaired t-test (C, D), significance is indicated as * p<0.05, ** p<0.01 and *** p<0.001.

During the early CG period, we expect animals to be hyperphagic when transferred to *ad libitum* food access. We first looked at the expression levels of *agouti related protein (agrp)* which is known to be an indicator of hunger in animals including zebrafish [62]. As expected we saw an increase in *agrp* expression in fish brains in the CR group compared to the DIO group, suggesting increased hunger in the calorically restricted group. When we tested CG9 fish one to two weeks after the transfer from CR to DIO conditions we found that *agrp* levels were significantly reduced compared to CR but had not yet returned to baseline compared to DIO (Fig 3A, ANOVA F(2, 6) = 114.4, p<0.0001). We measured food intake by counting living third instar larvae of *D*. *melanogaster*. Larvae can survive several hours in water without changing weight and continue to move which makes them an attractive prey for zebrafish. We first tested feeding in DIO and CR fish and found that food intake in DIO fish rises in proportion with body weight and therefore remains relatively constant if expressed in relation to body weight. At both timepoints tested, we found that CR fish when exposed to *ad libitum* feeding conditions are severely hyperphagic and eat nearly twice the amount of food as DIO fish (Fig 3B). The hyperphagia was significant at CG3 (Fig 3C, t(17) = 3.498, p = .0028) as well as CG9 (Fig 3D, t(18) = 3.622, p = 0.0020). This hyperphagia persisted for at least one week after the transition of CR fish into the obesogenic environment in both the CG3 (Fig 3C, t(16) = 6.150, p<0.0001) as well as the CG9 (Fig 3D, t(18) = 5.380, p<0.0001) groups.

### Adipose distribution in an obesogenic environment–the DIO phenotype

**Fig 4:**
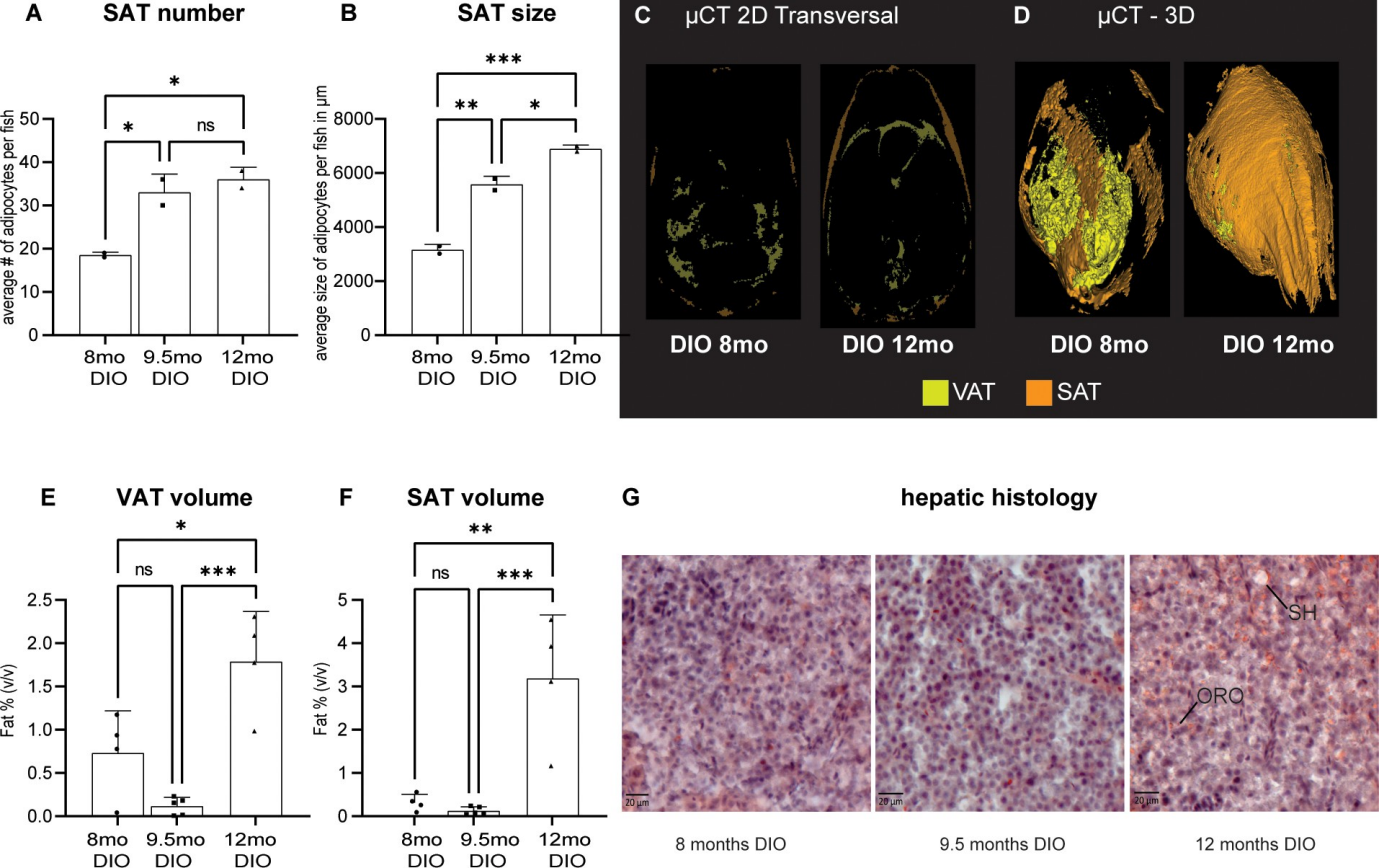
Adipose distribution under overfeeding conditions. (A) Subcutaneous adipose (SAT) cell number after 8 to 12 months of DIO; (B) average SAT adipocyte size after long-term DIO, Data from two representative male fish of the cohort; (C-F) Adult DIO fish of different ages and length on an obesogenic diet were compared for whole body adiposity using μCT imaging; (C) representative transverse section through DIO fish at 8 months and 12 months of age (D) representative three-dimensional volume rendering of visceral adipose tissue (VAT) and SAT; (E) quantification of VAT and (F) SAT, Data from 4–5 mixed gender fish and (G) representative hepatic histology of the 8, 9.5 and 12 month endpoints in DIO feeding conditions. Cryosections stained with hematoxylin for nuclei and oil red O (ORO) for lipids. Sections showed progressive signs of lesions indicative of steatohepatitis (SH). Error bars indicate STDEV, groups were compared with an ANOVA followed by Tukey’s multiple comparisons test, significance is indicated as * p<0.05, ** p<0.01 and *** p<0.001.

We first investigated adipose distribution in DIO fish. Evidence for the development of diet induced obesity has been shown in zebrafish for 8 weeks of overfeeding. However, the authors did not look at adipose distribution [35]. We carried out histology of DIO fish at 8, 9.5 and 12 months of age, a period where growth has ceased but which is associated with an increase in body circumference as zebrafish show positive allometric growth (Fig 2D). We saw that SAT adipocyte number increased between 8 and 9.5 months but not between 9.5 and 12 months of DIO (Fig 4A, ANOVA F(2,3) = 19.83, P = 0.0186). Adipocyte size increased between 8 and 9.5 months as well as between 9.5 and 12 months (Fig 4B, ANOVA F(2,3) = 144.9, P = 0.0010). This data shows that fish show hypertrophy across all three timepoints but gradually loose the capacity to store fat through adipocyte hyperplasia.

In order to get a better idea of the quantitative distribution of adipose tissue in zebrafish, we carried out μCT on adult DIO fish after increasing amount of time on the obesogenic diet (representative transverse section and 3D rendering see Fig 4C and 4D respectively). We quantified fat volume per skeletal segment across the body cavity and found that visceral adipose tissue (VAT) showed a significant increase in VAT at 12 months of DIO (Fig 4E, ANOVA F(2,10) = 17.76, P = 0.0005). We found the same increase in SAT in the 12 month DIO group (SAT Fig 4F, ANOVA F(2,10) = 18.56, P = 0.0004).

We tested liver sections for signs of fatty liver by staining cryo-sections with Oil red O and found evidence for increased hepatic adiposity between 8 months and 12 months of DIO. Further we saw early signs of steatohepatitis in the 9.5 and 12 months old hepatic tissue which we did not see at 8 months of age (Fig 4F). In our hands, the lipid droplets stained with oil red O (ORO) partly floated off and consequently not all lesions are positive for ORO.

### Adipose distribution–CG does not exacerbate the DIO phenotype

**Fig 5:**
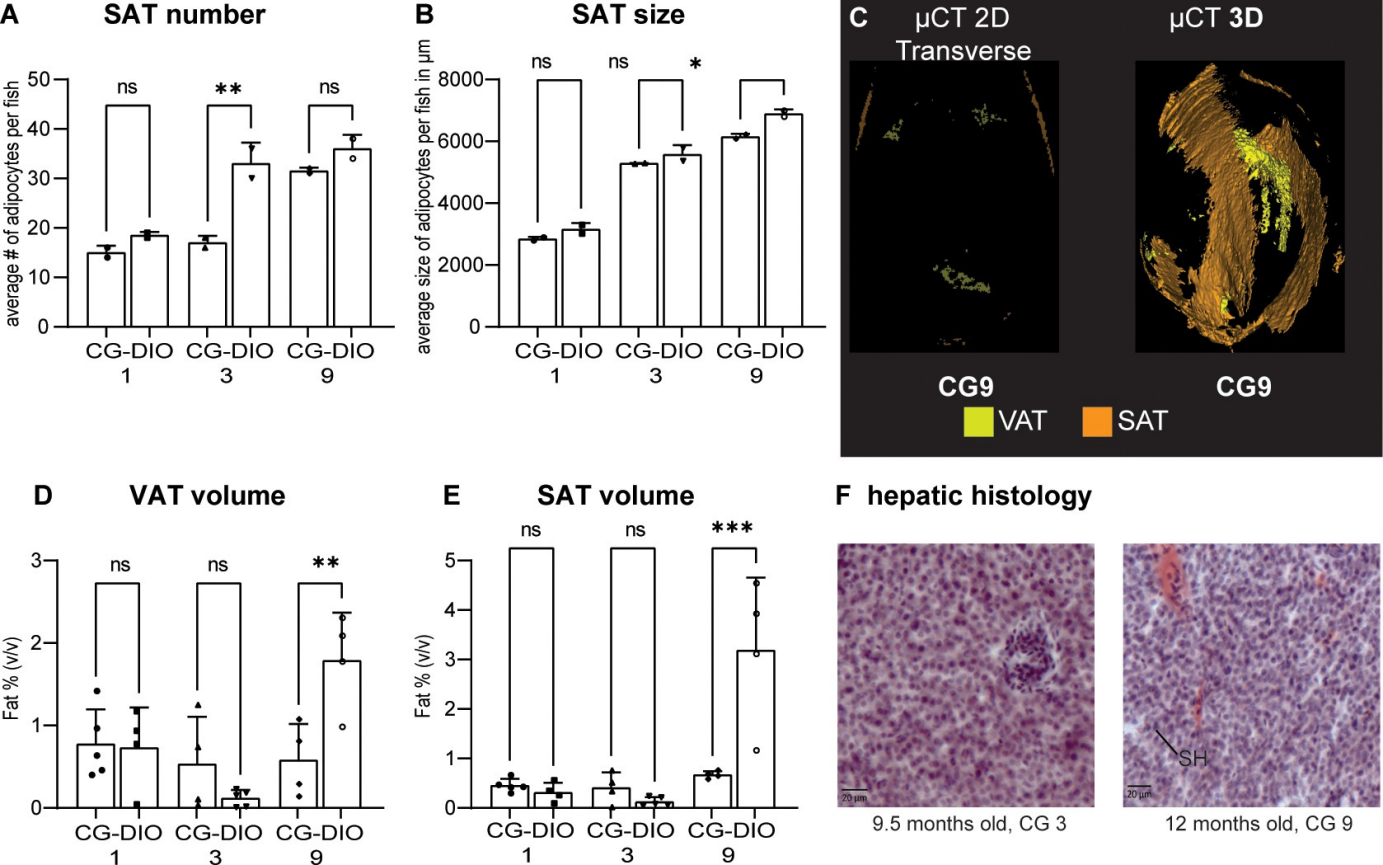
Early CG does not exacerbate DIO. Endpoint comparison of CG1, CG3 and CG9 animals with their respective DIO control groups at 8, 9.5 and 12 months respectively. (A) Subcutaneous adipocyte (SAT) number and (B) size. Data is from two representative male fish of the cohort; (C) CG1-9 fish were compared for whole body adiposity with DIO fish using μCT imaging; (D-E) quantification of visceral (VAT) and subcutaneous (SAT) adipose tissue by μCT, data from 4–5 mixed gender fish. (F) representative hepatic histology of the CG group stained with hematoxylin and oil red O (ORO). Sections show little ORO staining and few lesions (compare with Fig 4F). Error bars indicate STDEV, groups were compared with an ANOVA followed by Šídák’s multiple comparisons test, significance is indicated as * p<0.05, ** p<0.01 and *** p<0.001.

We next looked at whether CG exacerbates the DIO phenotype by both histology as well as μCT. Based on our histological examination of SAT, we found that the CG1 group catches-up in both adipocyte number as well as size while the CG3 group catches up in size but not number (Fig 5A adipocyte number ANOVA F(5,6) = 33.55, P = 0.0003 and Fig 5B adipocyte size ANOVA F(5,6) = 198.3, P<0.0001). Therefore, at an earlier timepoint, adipocyte hypertrophy is the predominant mode of compensation. At the later CG9 timepoint, the CG9 group catches up to the DIO group in number but not (quite) size of adipocytes (Fig 5A and 5B).

We also scanned CG animals with μCT (Fig 5C, compare to 4C) and found that VAT and SAT volume is caught up completely at the CG1 and CG3 timepoints but not the CG9 timepoint (VAT Fig 5C ANOVA F(5,20) = 6.564, P = 0.0009; SAT Fig 5D ANOVA F(5,20) = 15.57, P<0.0001). Staining hepatic tissue with ORO showed an increase in adipose deposition as well as steatic hepatosis in the CG9 group compared to the CG3 group but less adipose staining or lesions in the CG9 group compared to the DIO liver of the same age (Fig 5F, compare to 4G).

### CG in zebrafish—metabolic rates support a role for lean body mass in the late CG delay

**Fig 6:**
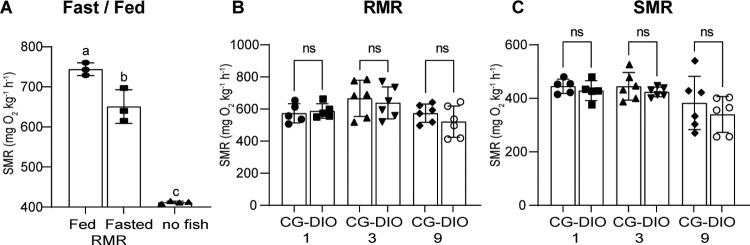
CG does not lead to changes in metabolic rate. (A) Shows the effect of fasting on resting metabolic rate in adult fish. RMR is lower during fasting conditions, n = 3 for fed and fasted, n of 4 for chambers without fish; (B) We compared CG1, CG3 and CG9 fish with their respective DIO control fish and found no difference in RMR; (C) We similarly found no difference in standard metabolic rate between the CG and the DIO control groups. n = 6 each, error bars indicate STDEV, groups were compared with an unpaired t-test (A) or an ANOVA followed by Šídák’s multiple comparisons test (B-C), significance is indicated as * p<0.05, ** p<0.01 and *** p<0.001.

We set-up intermittent respirometry to measure oxygen consumption in zebrafish and calculated metabolic rates. Oxygen consumption can be used to calculate routine and standard metabolic rate (RMR and SMR). We validated the procedure with fed and fasted animals since there should be a difference between these two groups due to the thermic effect of food. Consistent with previous publications [76–79], we found a significant increase in metabolic rate in fed fish compared to fasted fish. Respiration from bacteria (oxygen chambers without fish) was negligible (Fig 6A; ANOVA F(2,7) = 181.1, p<0.0001). When we looked at the CG groups, we found no difference in either RMR (Fig 6A; ANOVA F(5, 28) = 2.243, p = 0.0778) or SMR (Fig 6B; ANOVA F(5, 28) = 2.961, p = 0.0287) compared to the DIO groups.

### CG in zebrafish–role of mitochondria

**Fig 7:**
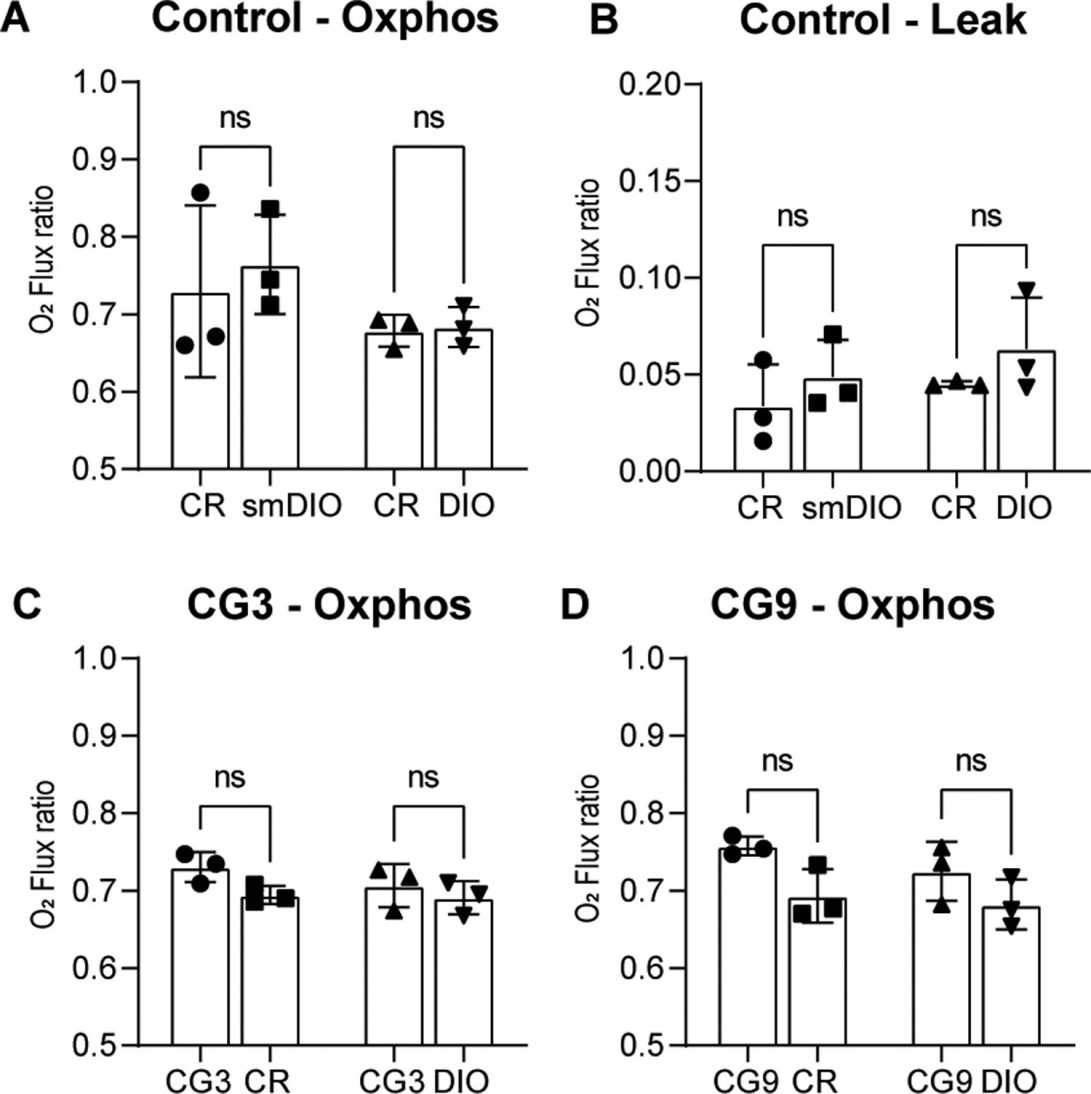
The shift to CG leads to an increase in maximal oxidative phosphorylation. (A) Due to the size difference between CR and DIO fish we tested age matched (CR and DIO) as well as size matched (CR and size matched (sm)DIO) maximal rate of oxidative phosphorylation and (B) the leakage of the mitochondrial membrane; (C-D) the maximal rate of oxidative phosphorylation in CG compared to control CR and compared to control DIO fish for (C) CG3 and (D) CG9; n = 3 for each group, error bars indicate STDEV, groups were compared with an ANOVA followed by Šídák’s multiple comparisons test.

We tested for mitochondrial function in CR and DIO fish. As there were significant size difference between CR and DIO fish we also included a size-matched DIO group which reached this size at a significantly younger age (1.5 months versus 9 months). Therefore, the CR and DIO groups are matched in age and the CR and size-matched DIO groups in size. We found that the maximal rate of oxidative phosphorylation did not differ between age matched or size matched calorically restricted or overfed fish (Fig 7A, ANOVA F(3,8) = 1.129, p = 0.3936). We next tested the leakage of the mitochondrial membrane which also did not differ between age matched or size matched CR and DIO fish (Fig 7B, ANOVA F(3,8) = 1.172, p = 0.3793).

When we compared the CG groups with the respective CR and DIO control groups, we found no evidence for changes in mitochondrial function at either CG3 (Fig 7C, ANOVA F(3,8) = 2.197, p = 0.1662) or CG9 (Fig 7D, ANOVA F(3,8) = 3.609, p = 0.0651). We saw a trend specifically in the CR to CG3 comparison with a t-test (t(4) = 2.766, p = 0.0505) which is significant in the CR vs CG9 group (t(4) = 3.034, p = 0.0386). Since we have multiple comparisons we used an analysis of variance followed by Šídák’s multiple comparison which is more stringent than an unpaired t-test. We found no differences between any of the groups in the mitochondrial membrane leak current (not shown).

### Traces of metabolic disease—glucoregulation

**Fig 8:**
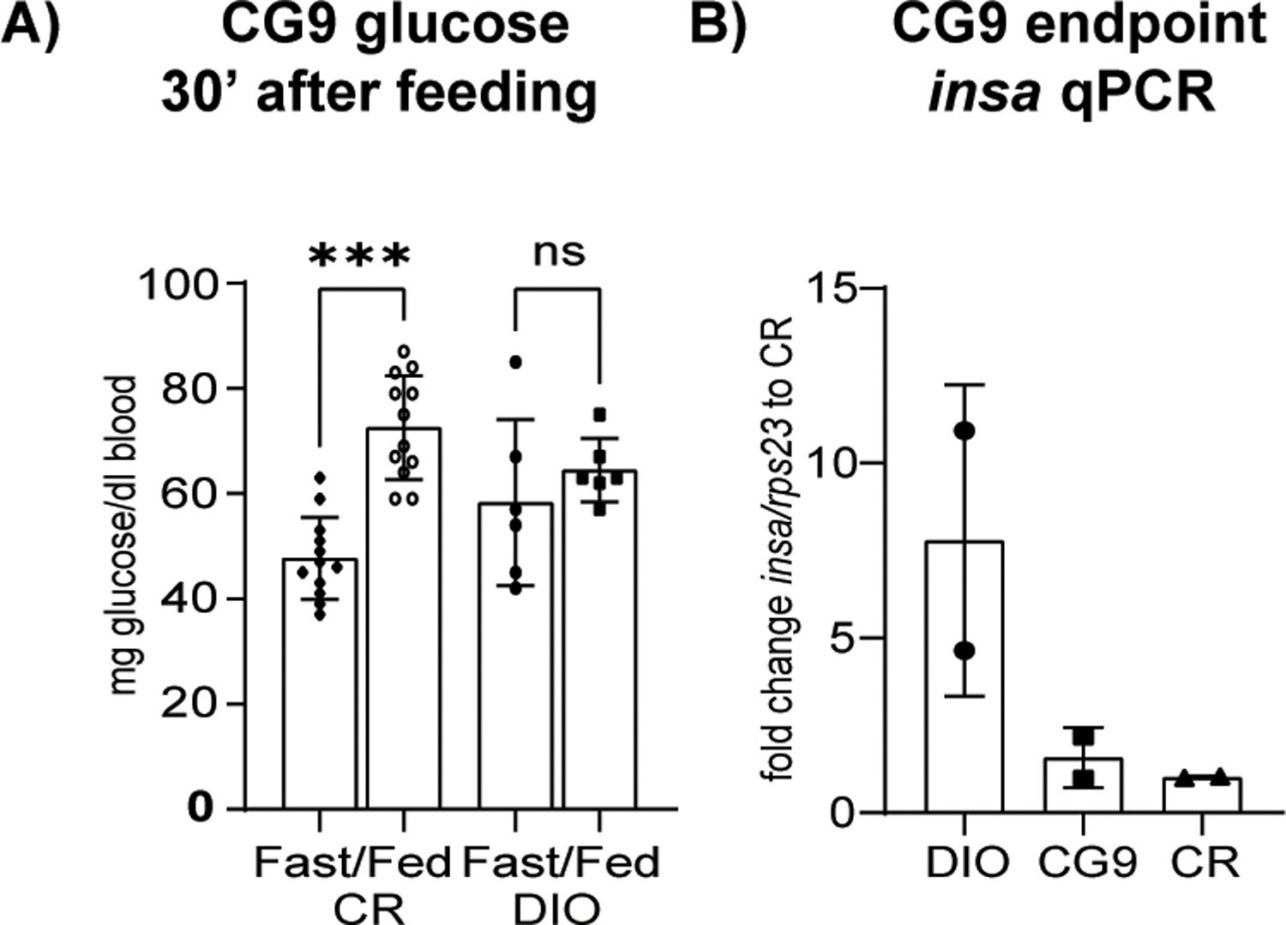
Glucoregulation at 9 months. (A) Blood glucose levels in fish fasted for 90 hours and tested 30 minutes after refeeding, CR n = 12 and DIO n = 6; (B) *insa* qPCR of whole fish body, n = 2; Error bars indicate STDEV, groups were compared with an ANOVA followed by Tukey’s multiple comparisons test (A) or Šídák’s multiple comparisons test (B-C), significance is indicated as * p<0.05, ** p<0.01 and *** p<0.001.

Elevated blood glucose levels at rest are an early sign of metabolic disturbance in obesity. We tested 9 months old fish fasted for 90 hours. We fed granulated food and tested blood glucose thirty minutes after feeding and found that CR fish respond with an elevated blood glucose level while DIO fish showed a reduced excursion (Fig 8A, ANOVA F(3, 32) = 2.075, p<0.0001). In order to support this data, we tested for insulin expression. In adult zebrafish, the islets are not in a distinct location but spread along the intestines. We therefore tested expression in whole bodies of fish. We only had two fish of the cohort for this analysis so the data is only indicative but we saw a trend towards an increased *insa* expression in DIO fish but not in CG9 fish compared to CR fish (Fig 8B, ANOVA F(2, 3) = 4.102, p = 0.1385).

## Discussion

Here we carried out a systematic characterization of growth responses in zebrafish. On one hand we aimed to establish whether we could observe obesity like endophenotypes in long-term overfed zebrafish, on the other hand we wanted to establish whether CG leads to an exacerbated DIO phenotype. Multiple authors have reported an increase in adiposity after overfeeding [35, 36], however long-term detrimental effects and markers are largely unknown. Further, there is controversy regarding genetic obesity models in the field such as the effect of leptin receptor mutations [30, 80]. Consequently, it is possible that fish (or given the wide range of life histories amongst fish at least zebrafish) store energy differently or at least not in form of adipose stores such as in mammals. In our previous study we showed that large adult females shunt energy primarily towards reproduction, followed by somatic growth and only then storage of fat [36]. Zebrafish are income breeders and as such given excess food could potentially increase daily reproductive capacity instead of storing energy for a later point [81]. This would circumvent increased adiposity and instead lead to increased body growth but no increase in body to mass index or conditioning index and we would expect no downstream deleterious effect of increased body adiposity such as lipotoxicity or hyperglycemia.

We therefore aimed to establish a more in-depth characterization of diet induced obesity in zebrafish. Linear regression of our growth curves showed that zebrafish undergo positive allometric growth. Positive allometric growth means that large specimens in the cohort show an increased circumference rather than an increased length. Therefore, at a higher SL, weight gain plays a predominant role while linear growth stagnates and/or plays a lesser role. From a mammalian and metabolic syndrome perspective, this is an interesting observation as obesity in mammals similarly is a lack of linear growth and instead a deposition of excess energy in adipose tissue. This data shows that while zebrafish grow linearly well into adulthood, this linear growth eventually stagnates and instead fish start depositing energy into circumference and likely adipose deposition. Looking at adipose depots in more detail, we found that SAT adipocyte number and size increased significantly over time, particularly at the late timepoint of 12 months. Further, SAT and VAT volume significantly increased in at 12 months of DIO but not before. Fish growth is sigmoidal with an early exponential phase, a linear phases and late growth stagnation. This suggests that energy allocation slowly shifts from linear growth to energy storage with a visible impact upon internal adipose tissue by one year of age. Further, previous studies have shown hepatic steatosis as a consequence of long-term overfeeding [35, 45, 46] which we were able to confirm in our long-term DIO fish which showed increased hepatic adipose deposition as well as increased number of hepatic lesions, which are reminiscent of the scarring seen in long-term obesity in mammals. When we looked at metabolic rate and mitochondrial function we did not see an overt phenotype in DIO animals. We saw a statistically not significant trend in the data comparing mitochondrial function between the CR and CG groups. A similar effect has been observed in mice during the first weeks after initiating a high fat diet where animals show changes in oxidative phosphorylation which normalize once animals adapt to the obesogenic diet [82]. For metabolic rate we looked at routine and standard metabolic rate (RMR and SMR). RMR is the energy required by an unfed animal which exhibits normal, spontaneous activity and is expected to be higher than SMR which is the energy needed to maintain proper body function [78, 83]. We found metabolic rate to be significantly reduced in DIO mammals due to the relative increase in adipose and decrease in muscle tissue. The lack of a difference between CR and DIO fish may be due to the fact that these animals are poikilothermic. The role of core temperature in obesity is controversial, but as a whole, obesity seems to lead to a reduced core temperature [84] similar to genetic obesity models [85]. In fish conversely, this variable is tightly controlled by the experimenter as core temperature is according to water temperature and the same across conditions. Further, a reduced temperature is known to reduce the metabolic rate. Lastly, we investigated glucose metabolism, specifically how fish responded to a food stimulus. Interestingly, DIO fish showed less of a glucose excursion 30 and 120 minutes after feeding compared to control animals suggesting an increased resorption of postprandial glucose from the bloodstream. In support of this, DIO fish showed an increased level of *insa* transcripts. This data suggests that fish undergo an adaptive response to overfeeding by increasing insulin production and thereby glucose resorption as opposed to an impaired glucose handling such as in evident in type 2 diabetes supporting previous evidence in zebrafish that oral glucose tolerance is impaired in long-term overfed fish [58]. It would be interesting to see whether specific strains of fish are more or less predisposed to DIO induced changes in glucose handling–adaptive as well as maladaptive as it is still largely unclear why some people with obesity respond with adaptive changes such as we saw in these fish and others develop type 2 diabetes. In conclusion, DIO zebrafish share hallmarks of long-term obesity with mammals. While the data presented here confirms that a long-term obesogenic environment leads to excess lipid deposition [35–38], hepatic lipid deposition and hepatic steatosis [35, 45, 46] as well as hyperglycemia and impaired glucose handling in zebrafish [38, 58], we did not find evidence for significant alterations in metabolic rate of mitochondrial function.

The other main direction of this study aimed to characterize CG in zebrafish. We wondered whether we could utilize the fish to model aspects of metabolic disease, particularly a predisposition to develop metabolic disease later in life after caloric restriction or undernourishment in early life / childhood. We found that body weight and length catch-up and do not compensate. Historically the two terms are often interchangeable, but Malcolm Jobling has pointed out key differences, specifically that compensation involves a period of increased growth rate above normal growth rates while catch-up growth involves animal growth at normal growth rates after a period of depressed growth [13]. Importantly, above-normal growth rates are associated with detrimental effects on animal physiology that could contribute to the aetiology of metabolic syndrome. Fast growth is correlated with increased oxidative stress [86], reduced protein turnover, increased telomere shortening, and leads to an accumulation of cellular damage and indeed reduced longevity [23, 87]. We did not observe increased growth rates after food restriction from 5dpf onwards. This was surprising to us as we initiated CG very early and had hypothesized that we would see compensatory growth. However, while growth rates in fish that were catching up increased upon transfer to *ad libitum* food conditions, they never exceeded those we saw in DIO conspecifics at a similar (earlier) period and standard length in life. This result suggests that either zebrafish do not respond with compensatory growth upon food restriction or that there is a critical period where growth stunting conditions lead to compensatory growth with an increased growth rate and that this period is already over 5 days post fertilization. Indeed, there is controversy in the mammalian literature about the effect of early growth restriction on compensatory growth and the most solid evidence involves food restriction during the peri-natal period, particularly by food restricting dams during pregnancy or pre-weaning [12]. In the medical literature, it appears that increased growth rates and downstream metabolic syndrome are mostly associated with pre-term birth and it has been suggested that the variable literature on catch-up growth in low weight infants is due to an unclear definition of low gestational age and compensatory growth is rather associated with pre-term birth when compared to term infants [1, 88]. Our study supports this notion as larvae feed off of maternally provided egg yolk until 5dpf, after which they need to provide sustenance themselves. Therefore, our experimental setup is analogous to caloric restriction immediately after a term birth / after weaning. Given the wealth of knowledge on zebrafish embryo development, it would be interesting to expand this study to look at the long-term effects of yolk depletion or maternal CR. Indeed, a recent study looked at partial yolk depletion in embryos and found changes in gene expression associated with metabolic syndrome at 2dpf [89]. However, the authors did not measure growth rates or characterized long-term responses between yolk depleted and sham treated siblings. This implies that malnutrition during these 5 days of yolk supplied energy could be comparable with pre-term birth and subsequent predisposition for metabolic syndrome. Therefore, it would be interesting to study which neural circuits are particularly predisposed to alteration by yolk depletion.

Based on the literature, one would predict that in the case of compensatory growth, the shift from CR to DIO would lead a long-term exacerbation of metabolic effects while in the case of catch-up growth one would expect effects similar to DIO or slightly behind the DIO group still catching up. Our growth curves support catch-up and not compensatory growth, and we indeed observed that none of the CG groups showed metabolic endpoints with an exacerbated phenotype compared to the DIO group. Indeed, some of the CG groups did not show a complete catch up process to the DIO group yet. While the early shift (CG1) caught up completely with the DIO group endpoints, the CG3 group mostly did and the CG9 group never caught up completely. The growth curves approximated each other, but especially in the metabolic endpoints there were still significant differences which are suggestive of a sequential process in the development of metabolic endpoints. For example, the CG3 group did catch up to the DIO3 group in adipocyte size but not number suggesting that SAT adipocytes undergo hypertrophy before the undergo a renewed period of hyperplasia. In mammals, hypertrophy during obesity is considered to be indicative of metabolic complications while hyperplasia is considered to be protective [43]. Fish appear to undergo both but sequentially. As far as overall levels of SAT and VAT around the body cavity are concerned, our μCT analysis showed that CG1 and 3 fish catch up to DIO fish but CG9 fish do not catch up to DIO9 fish in volume of adipose depots. This shows that adipose storage is a long-term process which specifically happens at a later timepoint of growth and that CG9 fish have not been in an obesogenic environment for long enough. Therefore, this data does not support that early lifetime caloric restriction (after 5dpf) is a risk factor for a later development and exacerbation of metabolic complications. Similarly, the hepatic steatosis we observed in the DIO9 groups could not be seen to the same extent in the CG9 group and the increased glucose resorption after feeding we saw in the DIO group but not the CG9 group suggesting that the islets had not undergone as significant an expansion as in the DIO9 group in order to compensate for the increased food intake. Therefore, the period of caloric restriction we utilized in these experiments was if anything protective of long term overfeeding induced effects and the CG9 fish never caught up to the DIO controls which were never privy to any food restriction. More studies would be needed as to whether maternal food restriction or overfeeding and/or food restriction before 5dpf would lead to long-term effects. This study lays the groundwork to explore this topic further.

In summary, it is notable that fish exhibit similar trends in the storage of lipids in an obesogenic environment compared to mammals and that fish of the Ekwill strain show several adaptive responses to DIO. However, significant variation exists between people in terms of response to obesity. Similarly, a recent paper showed differences between two strains of fish in fasting induced central gene expression changes [90]. It would be interesting to see whether similar strain differences exist in fish between metabolic endpoints such as a predisposition for adipose storage or a predisposition to type 2 diabetes. Further, while this study is used two large cohorts to establish the growth phenotype, we looked at metabolic markers (Figs 3–8) with a relatively moderate number of biological replicates. Obesity in mammals is known to induce relatively strong effects. We therefore decided to look for large biological effects in multiple metabolic markers. We saw such effects in the DIO groups compared to the CR groups. However, we did not see evidence that CG significantly exacerbates DIO (or that there is an observable trend in the data). Consequently, we cannot rule out that there are small biological effects in this data that would require significantly larger cohorts to establish. Interestingly, while there is controversy about the role of the leptin receptor in obesity in zebrafish [80] in studies that scored growth results before 6 months of age, a recent study showed that loss of the leptin b paralog in zebrafish leads to increased adiposity and a phenotype reminiscent of type 2 diabetes specifically in older fish between one and two years of age [91]. That is consistent with the data presented in this paper in that some metabolic endophenotypes only develop at a more advanced age. It would further be interesting to see whether infiltration of lipid into the liver, kidney, muscle or indeed the heart led to functional consequences for the zebrafish such as a reduced filtration rate or a reduced swim capacity.

## Supporting information

S1 Fig Compensatory growth can be induced by excessive feeding following caloric restriction: Cohort 2. (A, D) standard length; (B, E) fish weight and (C, F) body mass index of fish undergoing CG3 (A, B, C) of CG 9 (D, E, F); error bars indicate STDEV, ns indicates a lack of significant difference between the CG and the DIO group at that timepoint as indicated by a 2-Way ANOVA followed by Tukey’s multiple comparison test.
(TIF)

S2 Fig Compensatory growth (CG) can be induced by changes in feeding conditions and densities as well as with changes in feeding conditions only. (A,B) Growth curves of fish kept at different feeding conditions and densities; (A) Body lengths of fish raised in low density (5 fish per tank) but different feeding regimes; fish raised with ad libidum feeding conditions which is able to induce diet-induced obesity (DIO) show significantly increased linear growth compared to fish raised under caloric restriction (CR); fish raised under caloric restriction before 1 month of age but with ad libitum conditions afterwards (CG) show briefly increased growth rates and compensate for differences in body length suggesting that these fish exhibit compensatory growth (CG); (B) Body lengths of fish raised with different densities while every tank received the same amount of food resulting in different feeding conditions; fish raised in low density (5 fish per tank) with ad libidum feeding conditions (DIO) show significantly increased linear growth compared to fish raised in high density (50 fish per tank) and therefore under caloric restriction (CR); fish raised in high density (50 fish/tank, caloric restriction) before 1 month of age but in low density (5 fish/tank, ad libitum conditions) afterwards (CG) show briefly increased growth rates suggesting that these fish exhibit CG; n = 10 for each condition;; error bars indicate STDEV, ns indicates a lack of significant difference between the CG and the DIO group at that timepoint as indicated by a 2-Way ANOVA followed by a multiple comparison test.
(TIF)
