# Supplementary figures and images for: Long-term obesogenic diet leads to metabolic phenotypes which are not exacerbated by catch-up growth in zebrafish
Source: PLoS One. 2022 May 11;17(5):e0267933. doi: 10.1371/journal.pone.0267933 (PMC9094543; doi:10.1371/journal.pone.0267933)

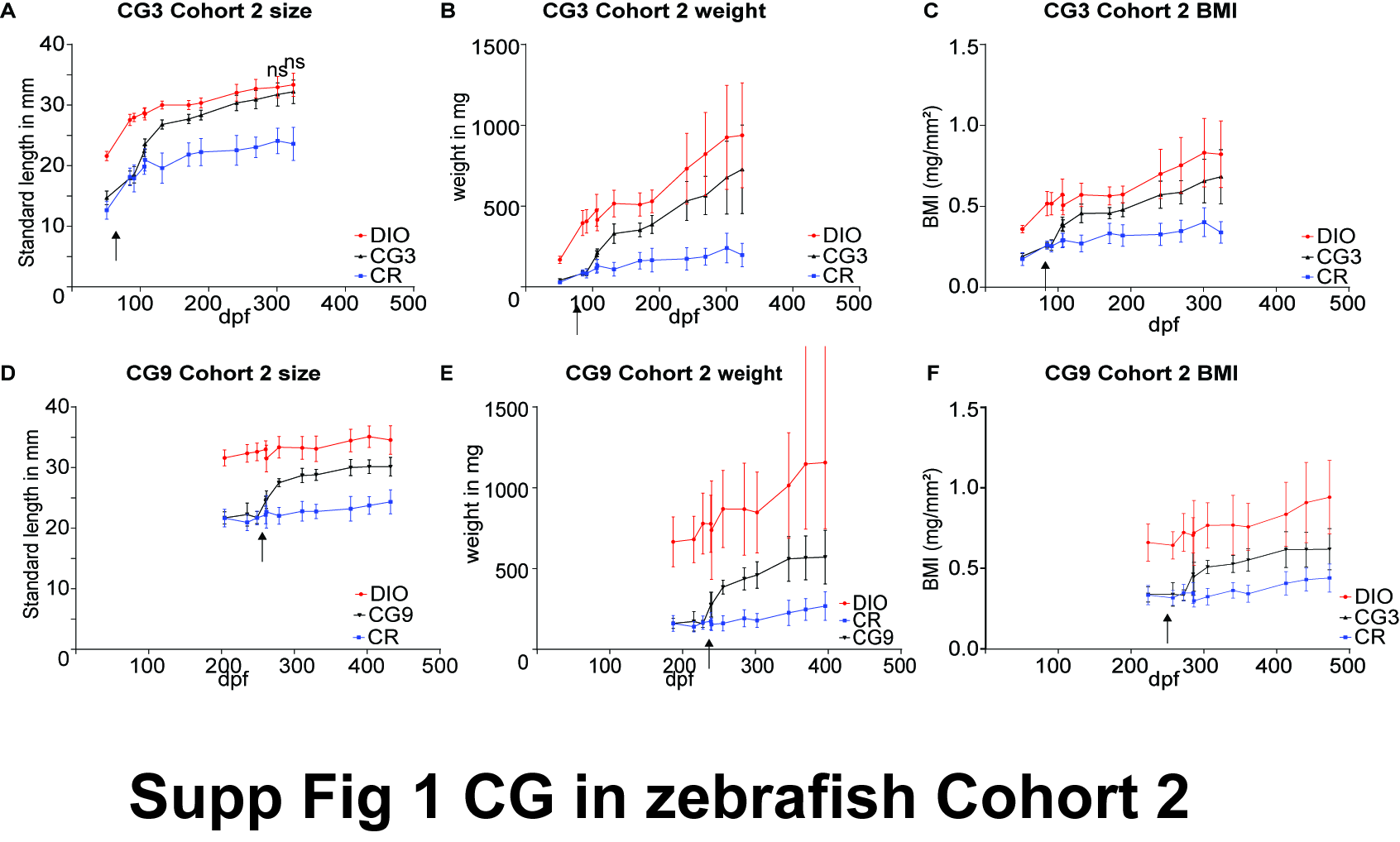

Supplement: S1 Fig — (A, D) standard length; (B, E) fish weight and (C, F) body mass index of fish undergoing CG3 (A, B, C) of CG 9 (D, E, F); error bars indicate STDEV, ns indicates a lack of significant difference between the CG and the DIO group at that timepoint as indicated by a 2-Way ANOVA followed by Tukey’s multiple comparison test. (TIF) [file pone.0267933.s001.tif]

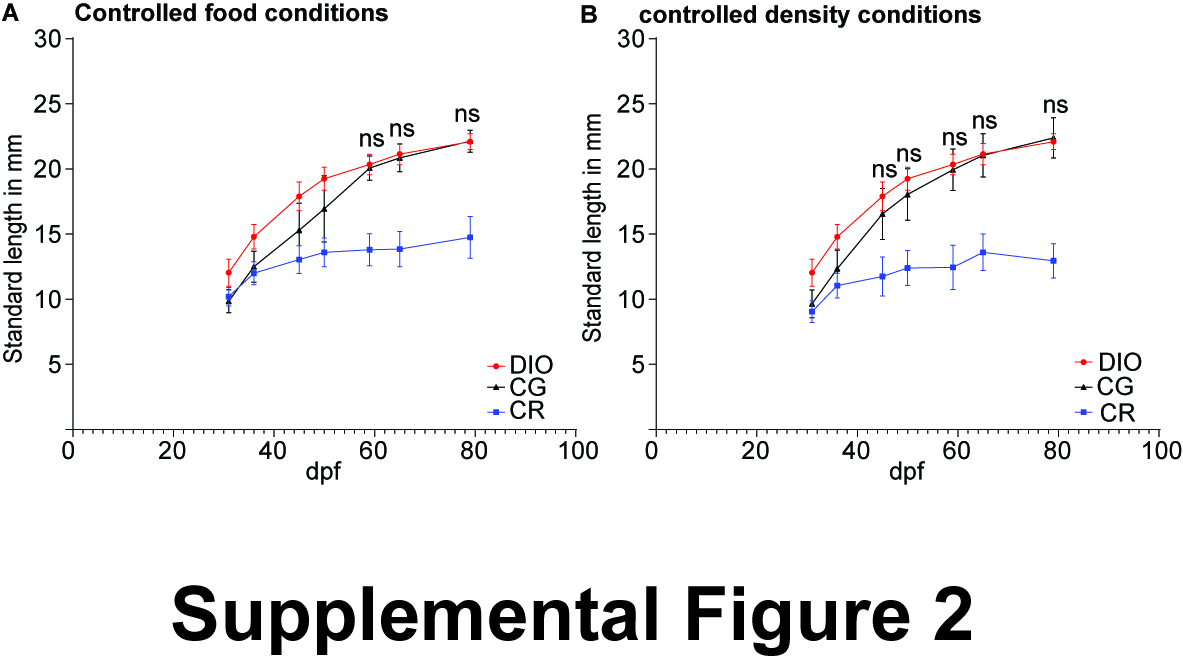

Supplement: S2 Fig — (A,B) Growth curves of fish kept at different feeding conditions and densities; (A) Body lengths of fish raised in low density (5 fish per tank) but different feeding regimes; fish raised with ad libidum feeding conditions which is able to induce diet-induced obesity (DIO) show significantly increased linear growth compared to fish raised under caloric restriction (CR); fish raised under caloric restriction before 1 month of age but with ad libitum conditions afterwards (CG) show briefly increased growth rates and compensate for differences in body length suggesting that these fish exhibit compensatory growth (CG); (B) Body lengths of fish raised with different densities while every tank received the same amount of food resulting in different feeding conditions; fish raised in low density (5 fish per tank) with ad libidum feeding conditions (DIO) show significantly increased linear growth compared to fish raised in high density (50 fish per tank) and therefore under caloric restriction (CR); fish raised in high density (50 fish/tank, caloric restriction) before 1 month of age but in low density (5 fish/tank, ad libitum conditions) afterwards (CG) show briefly increased growth rates suggesting that these fish exhibit CG; n = 10 for each condition;; error bars indicate STDEV, ns indicates a lack of significant difference between the CG and the DIO group at that timepoint as indicated by a 2-Way ANOVA followed by a multiple comparison test. (TIF) [file pone.0267933.s002.tif]
